# A contribution to MRI safety testing related to gradient‐induced heating of medical devices

**DOI:** 10.1002/mrm.29235

**Published:** 2022-03-28

**Authors:** Alessandro Arduino, Oriano Bottauscio, Mario Chiampi, Umberto Zanovello, Luca Zilberti

**Affiliations:** ^1^ Istituto Nazionale di Ricerca Metrologica (INRIM) Torino Italy

**Keywords:** gradient coil heating, medical devices, MRI safety, numerical simulation

## Abstract

**Purpose:**

To theoretically investigate the feasibility of a novel procedure for testing the MRI gradient‐induced heating of medical devices and translating the results into clinical practice.

**Methods:**

The concept of index of stress is introduced by decoupling the time waveform characteristics of the gradient field signals from the field spatial distribution within an MRI scanner. This index is also extended to consider the anisotropy of complex bulky metallic implants. Merits and drawbacks of the proposed index of stress are investigated through virtual experiments. In particular, the values of the index of stress evaluated for realistic orthopedic implants placed within an ASTM phantom are compared with accurate heating simulations performed with 2 anatomic body models (a man and a woman) implanted through a virtual surgery procedure.

**Results:**

The manipulation of the proposed index of stress allows to identify regions within the MRI bore where the implant could affect the safety of the examinations. Furthermore, the conducted analysis shows that the power dissipated into the implant by the induced eddy currents is a dosimetric quantity that estimates well the maximum temperature increase in the tissues surrounding the implant.

**Conclusion:**

The results support the adoption of an anisotropic index of stress to regulate the gradient‐induced heating of geometrically complex implants. They also pave the way for a laboratory characterization of the implants based on electrical measurements, rather than on thermal measurements. The next step will be to set up a standardized experimental procedure to evaluate the index of stress associated with an implant.

## 1 INTRODUCTION

MRI diagnostics for patients carrying medical devices may involve safety hazards that have to be properly addressed. The relevance of this problem is growing because of the increasing number of citizens carrying implants and, also considering comorbidity effects, because of the increase in the probability for elderly people to both need an implant and an MRI scan. Available data show that, in the European Union, ∼50 million citizens carry some kind of implant and, in the age group between 60 and 80 years, ∼50% of them need an MRI scan during the lifetime of their implant.^1^, ^2^, ^3^

Demonstrating compliance with MRI safety and getting the “MR conditional” labeling is a challenging and costly process for implant manufacturers.^4^ This problem is also relevant for MRI scanner producers, who have to improve the device safety for handling the exposure of patients with an implant.

When scanning a patient with an implant, one of the main safety issues is given by the MR‐induced heating of the tissues surrounding the metallic parts of the implant. Significant progress in the analysis of this kind of risk was achieved in the last decade, but the process is far from being completely settled.^5^, ^6^ The literature on radiofrequency (RF) heating of metallic implants is nowadays quite wide, exploring most of the critical issues arising in clinical scanners, both for passive^7^, ^8^, ^9^, ^10^, ^11^, ^12^, ^13^ and active^14^, ^15^, ^16^, ^17^, ^18^ implants. It is often assumed implicitly that the MR‐induced heating in presence of implants is because of the RF field,^19^, ^20^, ^21^ but recently an additional hazard caused in bulky passive implants (like joint replacements) by the exposure to MRI gradient fields has been shown.^22^, ^23^, ^24^, ^25^, ^26^, ^27^, ^28^

The RF heating is induced directly in the biological tissues by the electromagnetic field, whose spatial distribution can be severely affected by the presence of metallic implants. On the other hand, the gradient field may induce significant eddy currents and Joule losses only within the implant, which heats up and successively diffuses the heat toward the biological tissues.

The current state of the art implant safety can be summarized with reference to the standards ISO/TS 10974 on active implants^29^ and ASTM F2182 on RF‐induced heating of passive implants,^30^ which are periodically updated to account for the new findings coming from the scientific literature.^31^

The ASTM F2182 document describes a standard test method that covers RF‐induced heating on or near a passive medical implant within a phantom undergoing magnetic resonance imaging.

The standard ISO/TS 10974 is applicable to active implantable medical devices in patients who undergo an MR scan in 1.5T whole body scanners. It covers protection from harm caused by RF‐induced heating and gradient‐induced heating, as well as gradient‐induced vibrations, B_0_‐induced forces and torques, gradient‐induced extrinsic electric potentials and possible malfunctions. Regarding the gradient‐induced heating hazard, ISO/TS 10974 requires that the device under test is oriented and positioned inside the bore to maximize the root mean square of the time derivative of the gradient field component orthogonal to the largest conductive surface of the active implantable medical device. Exceptions are considered when the device has a complex shape. In this case, the orientation at which the maximum heating occurs must be identified experimentally. The gradient waveform to be used in the testing is selected according to a 2‐tier approach, with a Tier 1 using a standardized waveform and a Tier 2 using clinical waveforms. The test procedure is based on the measurement of the temperature hot spot on the surface of the metallic object immersed in a gelled solution. In the case of devices with a complex shape, it can be difficult to find the location of the main hot spot.

Because of the suggested conservative approach, based on the worst orientation and positioning of the implant within the scanner, the extension of the ISO/TS 10974 to bulky orthopedic implants could lead to excessively strict conditions. Although for many active implantable medical devices it is reasonable to assume a complete lack of knowledge about the implant orientation, large orthopedic implants like hip, knee and shoulder joints are inserted in the body with a specific orientation, which is known with respect to the scanner reference system up to a reasonable uncertainty.

To address this issue, this paper proposes an anisotropic index of stress that, taking into account the (possibly partial) knowledge of the orientation of bulky orthopedic implants, provides a less conservative condition than that obtained from a direct extension of the ISO/TS 10974. In particular, a simple hardware‐agnostic procedure for testing these implants can be devised, both addressing the needs of testing labs and keeping the capability to translate the testing results into a real clinical scenario. This allows overcoming another limitation of the ISO/TS 10974, which currently prescribes to perform the tests using tabulated values of the root mean square of the time derivative of the magnetic field generated by the gradient coils. However, these values in the future could be no longer representative, considering the development of new technologies^32^ and the introduction of new sequences,^33^ therefore provoking the need for a continuous update of the standards in order to keep trace of the new reference values.

## 2 THEORY

**FIGURE 1:**
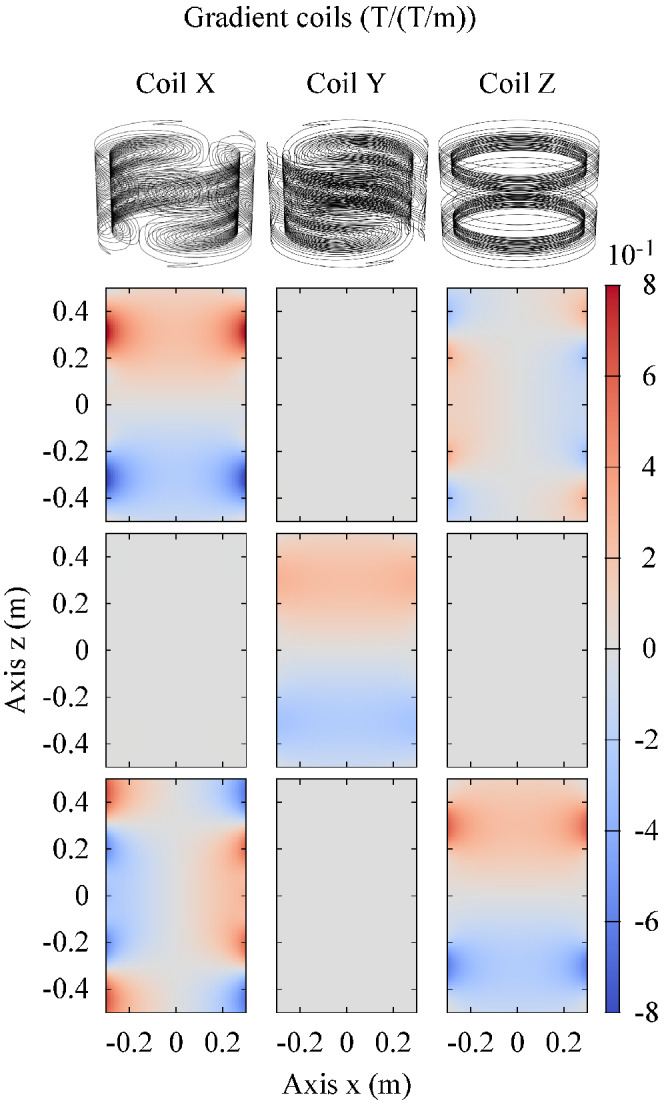
Spatial distribution in the plane *y* = 0 of the magnetic field components produced by tubular gradient coils with a unit gradient (1 T/m) around the isocenter. The first row depicts the *x* components, the second row the *y* components and the third row the *z* components

**FIGURE 2:**
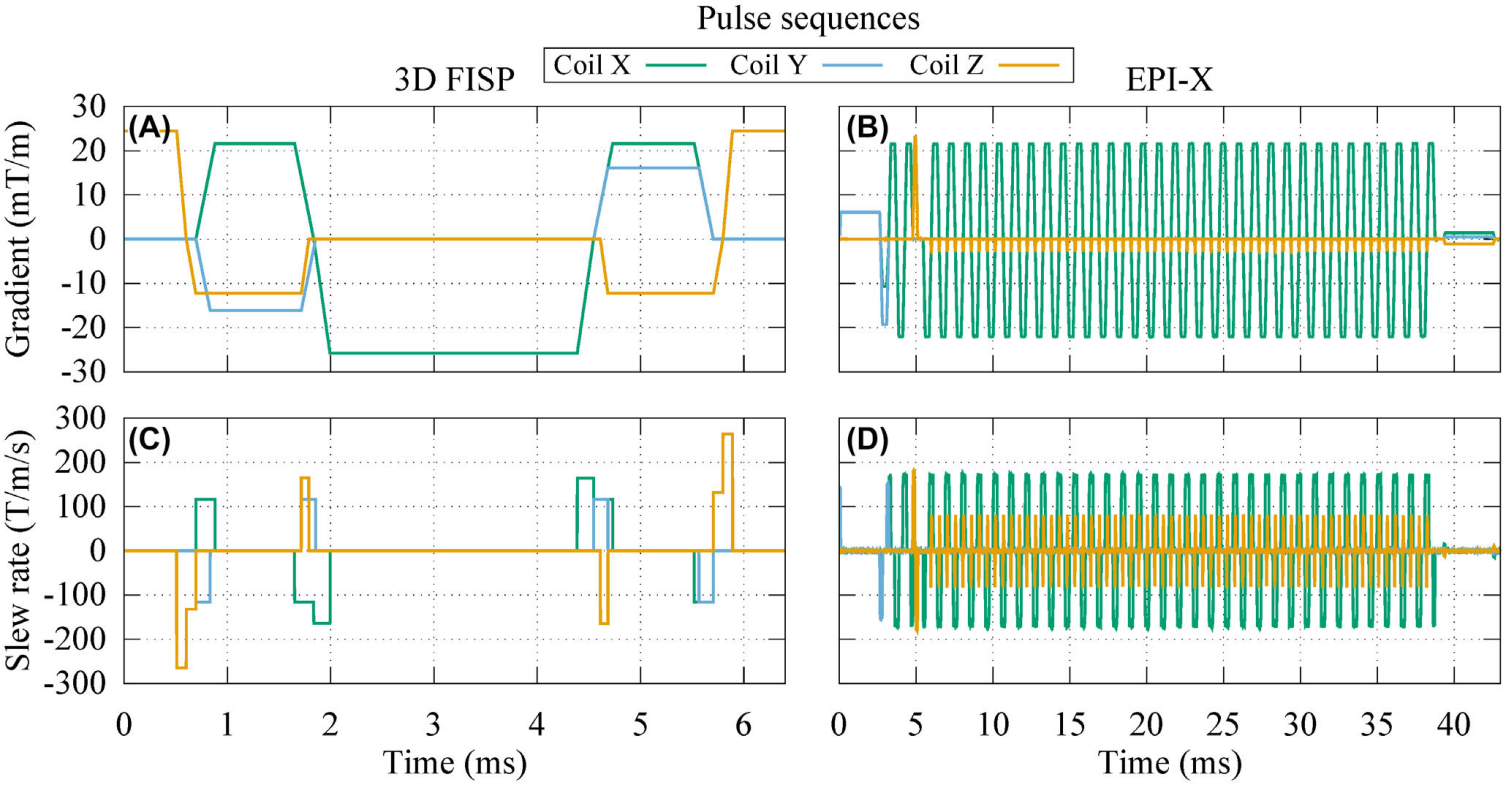
Waveforms of the gradients produced by each gradient coil during 1 repetition time of a 3D FISP or an EPI‐X pulse sequence with the frequency encoding signal associated to the coil X (A,B) and their time derivatives (C,D)

Let **B**_*i*_(**x**) denote the magnetic field produced by the *i*‐th gradient coil (GC) when the gradient at the isocenter is 1 T/m along the coil reference direction (Figure 1). The time evolution of the magnetic field produced in **x** by the considered system of GCs powered according to a given pulse sequence is

(1) $$B\left(x,t\right)=\sum_{i=1}^{3}G_{i}\left(t\right)B_{i}\left(x\right),$$

where *G*_*i*_(*t*) is the waveform of the gradient, expressed in T/m, produced by the *i*‐th GC during the application of the pulse sequence (Figure 2).

It is well known that, for frequencies low enough to not incur in the skin effect, the energy density deposited by a time‐varying magnetic field within a metallic object through the induced eddy currents is proportional to^29^

(2) $$\begin{array}{cc} & I\left(x\right)=\frac{1}{T}\int_{0}^{T}{\left\|\frac{\partial B}{\partial t}\left(x,t\right)\right\|}^{2}dt, \end{array}$$

where *T* is the period, or the repetition time, of the pulse sequence. This averaging is justified by the different time‐scales of the electromagnetic and thermal phenomena^28^, ^34^ and it is consistent with the use of the root mean square of the time derivative of the magnetic field as a reference test value in ISO/TS 10974.

Therefore, if **B**(**x**,*t*) is the magnetic field generated by the GCs during the execution of a given pulse sequence of length *T*, the quantity *I*(**x**) is an index of the thermal stress that the metallic implant experiences if located in **x**. In this case, the separation of quantities provided in Equation (1) is used to elaborate Equation (2) obtaining

(3) $$\begin{array}{cc} & I\left(x\right)=\sum_{i=1}^{3}\sum_{j=1}^{3}\underset{Q_{\mathrm{ij}}}{\underbrace{\frac{1}{T}\int_{0}^{T}\frac{dG_{i}}{dt}\left(t\right)\frac{dG_{j}}{dt}\left(t\right)\;dt}}\;B_{i}\left(x\right)\cdot B_{j}\left(x\right). \end{array}$$

Here, the symmetric and positive semi‐definite matrix **Q** collects all the information related to the adopted pulse sequence, or, more precisely, to its slew rates.

In the definition of the index of stress *I*(**x**) provided by Equation (3), all the directions along which the magnetic field could be oriented are weighted in the same way, leading to an isotropic index of stress meaningful only for a spherical elementary volume, in which the magnetic field is assumed to be uniform. In the case of a real implant with a complex shape, *I*(**x**) cannot discern the actual implant orientation. As such, its limitation in a safety perspective can only lead to a conservative approach analogously to the testing procedure described by the ISO/TS 10974. To take into account the anisotropy of a real orthopedic implant, a unitless weighting coefficient *η*_**u**_ can be associated to each direction **u** along which the magnetic field produced by the GCs could be oriented. The anisotropic index of stress associated to the implant is then written as

(4) $$\begin{array}{cc} & I_{\text{aniso}}\left(x\right)=\sum_{i=1}^{3}\sum_{j=1}^{3}Q_{\mathrm{ij}}\;\eta_{B_{i}\left(x\right)}\;\eta_{B_{j}\left(x\right)}\;B_{i}\left(x\right)\cdot B_{j}\left(x\right). \end{array}$$

Here, the field is assumed to be uniform within the implant volume.

## 3 METHODS

### 3.1 Definition of the weighting coefficient

The weighting coefficient *η*_**u**_ that appears in Equation (4) is determined through an experimental characterization of the bulky metallic implant under test. A dosimetric quantity *D* is assumed as representative of the thermal stress and measured experimentally when the implant is subjected to a low frequency uniform magnetic field with a given intensity oriented along the direction **u**. The choice of a low frequency field (on the order of some hundreds hertz at most, close to the 270 Hz frequency of the sinusoidal waveform for Tier 1 in the ISO/TS 10974) avoids the onset of the skin effect for the alloys commonly adopted in implants.

Repeating the measurement for a certain number of directions, a function *D*(**u**) is obtained by interpolating the measured quantities. Therefore, the weighting coefficient *η*_**u**_ is defined as

(5) $$\begin{array}{cc} & \eta_{u}=\sqrt{\frac{D\left(u\right)}{\max_{v}D\left(v\right)}}, \end{array}$$

assuming that the dosimetric quantity depends quadratically on the magnetic field intensity. The weighting coefficient so defined belongs to the interval [0,1], so that the anisotropic index (Equation [4]) is always less than or equal to the isotropic one (Equation [3]). In particular, the indexes are equal only when the field orientation is associated with the maximum value of the dosimetric quantity.

Possible choices for the dosimetric quantity, depending quadratically on the magnetic field intensity, are: the local peak of the volume power density averaged over the period *T* (*p*_max_); the total power deposited in the implant averaged over the period *T* (*P*); the local peak of the temperature increase after 360 s (Δ*T*_max,360s_) or after 900 s (Δ*T*_max,900s_). Other physical quantities could be identified to fulfill this purpose.

Measurements are performed using a phantom with the features described in ASTM F2182. In this case, the phantom container is a parallelepiped of sizes 650 mm × 420 mm × 90 mm, filled with a saline gel. The thermal properties of the gel are: thermal conductivity equal to 0.54 W/(m K), specific heat capacity equal to 4152 J/(kg K), mass density equal to 998 kg/m^3^.

In this paper, as a proof of concept, laboratory experiments are replaced by virtual experiments, where simulations are performed using computational tools.^28^ The adopted virtual model consists of the studied implant (hip, knee, or shoulder), with metallic parts made of CoCrMo alloy and without porous or ceramic coating, placed inside the phantom. All the models, presented in Table 1, are discretized with a uniform voxel mesh of 2 mm.

**TABLE 1:** Description of the simulated implants and anatomic human model where they are located

Implant	Model	Description	3D view
Hip	Glenn	140 mm long stem Hemispherical head of diameter 34 mm Acetabular shell 5 mm thick and diameter 54 mm 8.5 mm thick liner	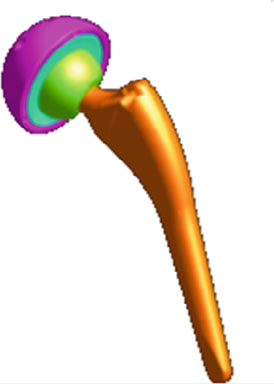
Hip	Yoon‐Sun	125 mm long stem Hemispherical head of diameter 28 mm Acetabular shell 3.5 mm thick and diameter 42 mm 3.8 mm thick liner	
Knee	Glenn	Tibial component of maximum size 77 mm Femoral component of maximum size 77 mm	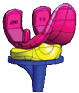
Knee	Yoon‐Sun	Tibial component of maximum size 66 mm Femoral component of maximum size 66 mm	
Shoulder	Glenn	115 mm long stem Hemispherical head of diameter 35 mm	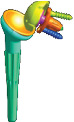
Shoulder	Yoon‐Sun	105 mm long stem Hemispherical head of diameter 40 mm	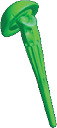

*Note*: The metallic parts are always assumed to be made of CoCrMo alloy without porous or ceramic coating.

The simulation results have been validated by comparison with experimental measurements performed with realistic setups. In previous publications,^28^, ^35^ the heating of the acetabular cup of a hip implant exposed to the field generated by a GC supplied with a trapezoidal waveform has been recorded and compared with simulation results. Analogous comparisons for the knee and the shoulder implants are reported in the Supporting Information Appendix A. Different orientations of the implants with respect to the GC system are considered, highlighting the effect of anisotropy.

**FIGURE 3:**
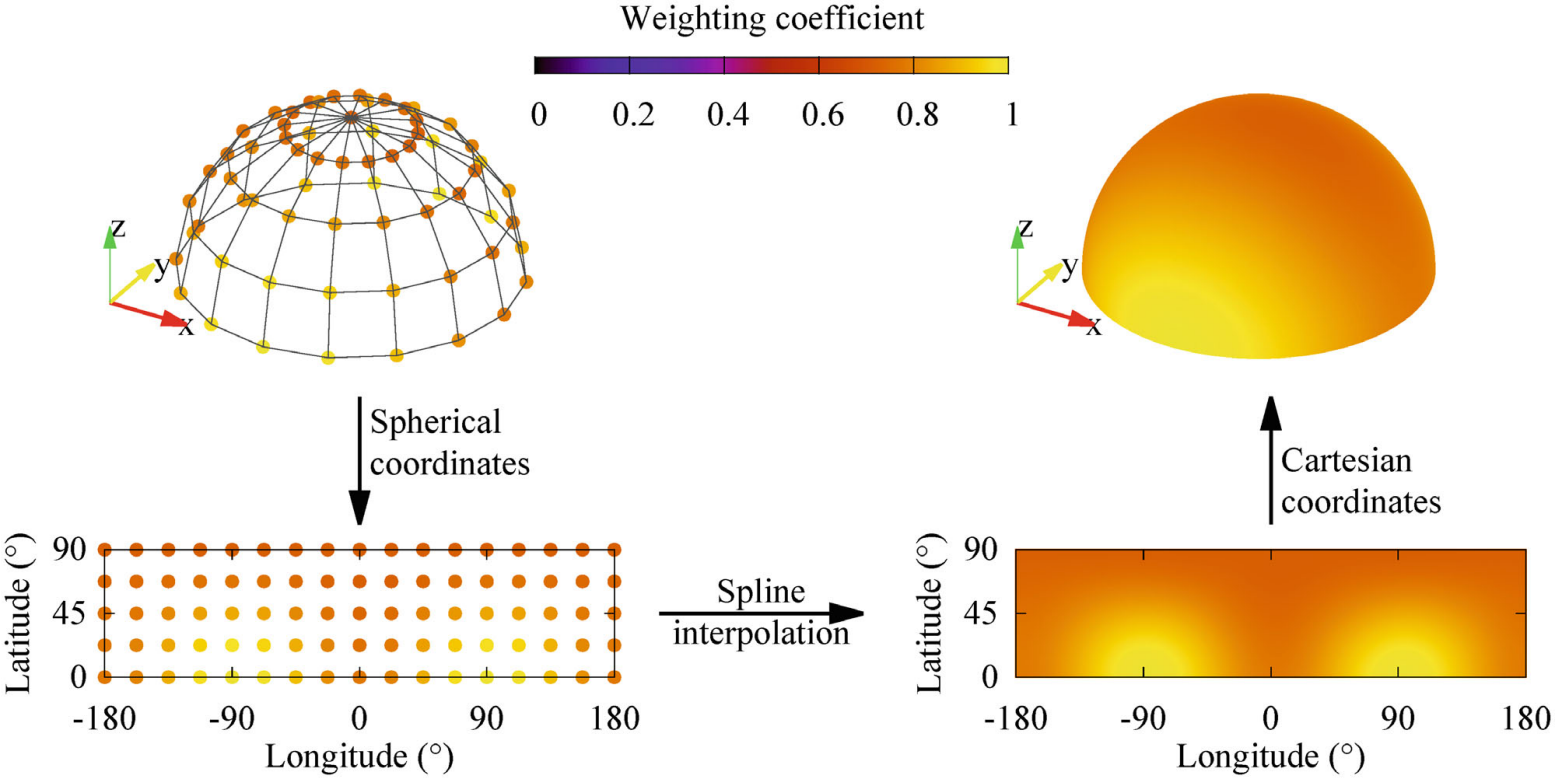
Sketch of the procedure for the evaluation of the weighting coefficient associated with a generic direction of the magnetic field starting from a limited number of measured samples. The set of the admissible directions coincides with a hemisphere, where each direction is identified by a segment OP from the center of the hemisphere (O) to a point on the hemisphere (P). The hemisphere is described in spherical coordinates, in which the latitude is the angle between OP and its projection on the plane *z* = 0, whereas the longitude is the angle between the projection of OP in the plane *z* = 0 and the *x*‐axis. The reported weighting coefficient is the one obtained for the hip implant of Yoon‐Sun model with total power as dosimetric quantity

To estimate the weighting coefficient, the following remarks have to be considered. The minimum set of admissible directions of the magnetic field with respect to the implant is represented through a hemisphere. Precisely, each direction of the magnetic field is identified by a segment OP connecting the center of the hemisphere (O) to a point on the hemisphere (P). The adoption of a spherical coordinate system allows to identify each direction in the hemisphere with a longitude (between −180° and 180°) and a latitude (between 0° and 90°). Because any reversal of the magnetic field with respect to the equatorial plane would not change the dosimetric results, the complementary hemisphere (latitude between 0° and −90°) is of no interest. The experiments are conducted with magnetic field orientations derived from a structured mesh of longitudes and latitudes. The measured weighting coefficients can be easily extended to any other direction by interpolation on the plane of longitudes and latitudes, for example using a spline interpolator. This procedure is sketched in Figure 3.

In the following, for each considered implant, 5 latitudes and 16 longitudes are simulated in the preliminary virtual experiments, as reported in Figure 3 for the hip implant. This discretization determines a grid with resolution of 22.5° along both angles, representing a good trade‐off between spatial reconstruction and number of experiments. A similar outcome could be produced by laboratory experiments setting up the procedure to get all these measurement data properly.

### 3.2 Determination of the maximum admissible index of stress

The maximum admissible index of stress for the implant under analysis is determined reusing the same measurements performed to estimate the weighting coefficient. In this case, the implant is exposed to a simple sinusoidal magnetic field

(6) $$\begin{array}{cc} & B\left(t\right)=B\sin\left(\omega t\right)\hat{u}, \end{array}$$

of amplitude *B* and angular frequency *ω*. The related index of stress defined in Equation (3) is

(7) $$\begin{array}{cc} & I=\frac{B^{2}\omega^{2}}{2}. \end{array}$$

This index of stress is associated with the maximum value measured for the dosimetric quantity *D* and scales linearly with it, being the skin effect negligible in the range of frequencies of the gradient fields. Therefore, for a given safety limit of the dosimetric quantity, the corresponding safety limit of the index of stress computed in Equation (7) can be evaluated by rescaling.

**FIGURE 4:**
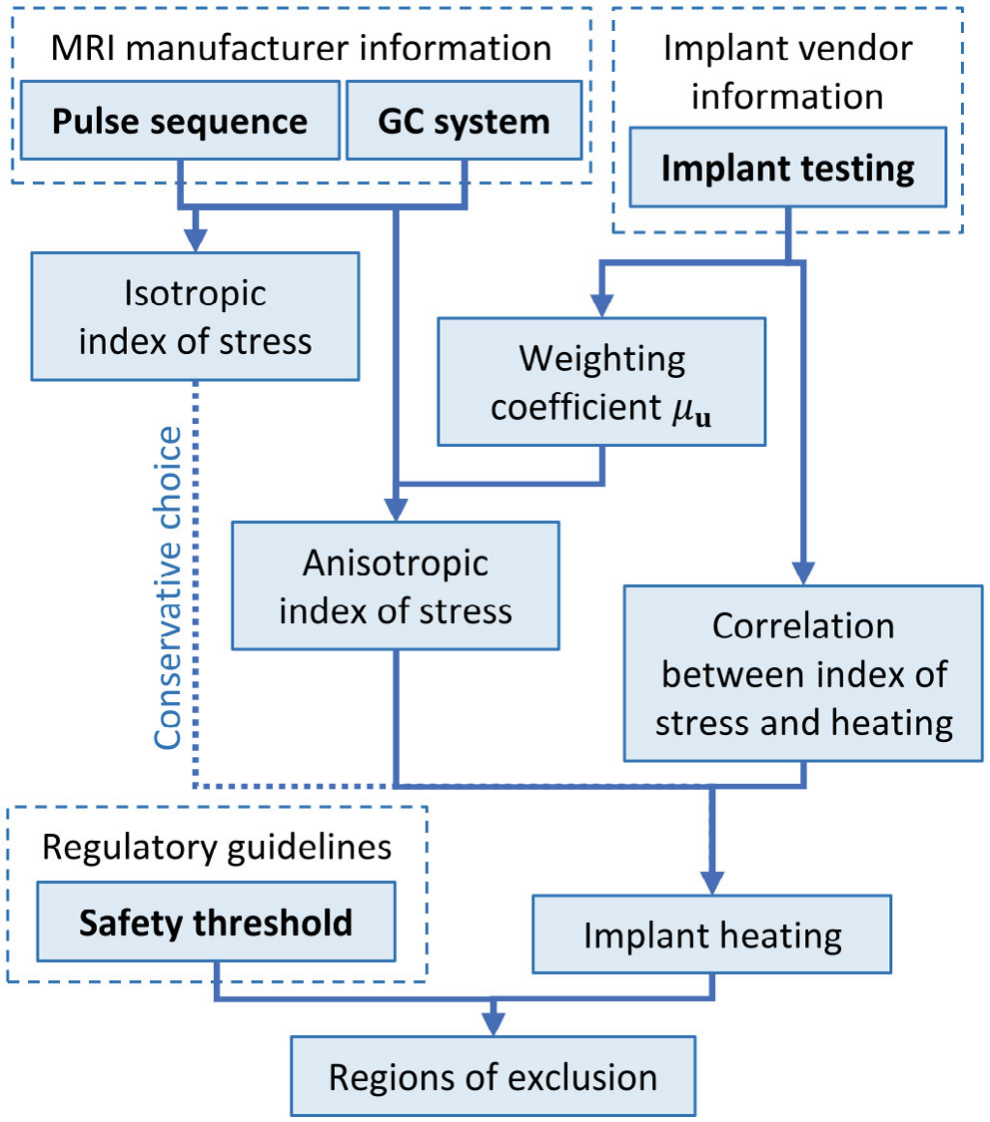
Schematic summary of the proposed procedure. The input data are in bold

In the following, a peak temperature increase after 900 s of 1 K on the implant surface is assumed as safety limit. This arbitrary choice can be changed with respect to both the reference dosimetric quantity and the safety limit without invalidating the procedure. The limit on the index of stress is obtained by rescaling it directly with reference to the temperature increase observed in the simulated phantom. The comparison between the index of stress obtained for an imaging procedure in presence of the implant and the identified limit determines if the implant position falls in a region of exclusion for the imaging procedure, as sketched in Figure 4. An accurate comparison should include a correction coefficient that translates the result from in vitro to in vivo (see below). Moreover, when the total power deposited in the implant (or, alternatively, the peak of power density) is used as a dosimetric quantity, a correlation coefficient that relates the deposited power to the temperature increase is needed.

### 3.3 In vivo versus in vitro results

The results obtained using phantom experiments cannot be directly taken as an assessment of in vivo situations (e.g., Guo et al, Guo et al, and Fiedler et al).^36^, ^37^, ^38^ This problem is particularly complex for RF exposure, when both electrical and thermal properties of tissues affect the heating. Gradient‐induced heating, instead, originates in the implant independently of the low conductivity of the tissues. Therefore, its diffusion toward the surrounding tissues is determined only by their thermal properties. The main issues in translating results obtained in a phantom to in vivo results are related to the subject‐specific patient anatomy, the actual thermal properties of the biological tissues, and the thermoregulation effects, like blood perfusion. Despite correcting the phantom measurements to reflect the in vivo behavior is far from the scope of this article, a correlation between the 2 situations is estimated by comparing numerical results.

To this purpose, the Glenn and Yoon‐Sun anatomic models (Virtual Population: Zurich MedTech AG, Zurich, Switzerland)^39^ are considered. Glenn is an 84‐year‐old adult male (height, 1.73 m; weight, 61.1 kg), composed of 72 different biological tissues, whereas Yoon‐Sun is a 26‐year‐old adult female (height, 1.52 m; weight, 54.6 kg) composed of 71 different biological tissues. Both models are discretized with a uniform voxel mesh of 2 mm. Electrical and thermal properties are taken from the IT'IS Database.^40^ Non‐linear thermoregulation effects for blood perfusion and metabolic heat are included in the model.^28^

The 2 models received a virtual surgery to implant the most appropriate hip, knee, and shoulder prosthesis without porous or ceramic coating and the metallic parts made of CoCrMo alloy (Table 1). To reproduce a realistic surgery, some pieces of the bones are severed. Where the implant does not replace the removed tissues perfectly, a filling material with the properties of connective tissue or synovial fluid (assumed equal to those of the cerebrospinal fluid) is inserted.

For each implanted anatomic model, 2 sets of simulations are performed, considering the implant immersed in an ASTM phantom or inserted in the anatomic model with the virtual surgery. The position of the implant with respect to the MRI system is the same in the 2 cases. In the case of the phantom, the latter is centered around the implant (the heating is localized in close proximity to the implant, its relative positioning with respect to the phantom does not affect the result). For each set, 9 positions of the implant within the scanner are considered to experience different gradient field amplitude and directions. At each position, 4 pulse sequences are simulated: a 3 dimensional (3D) fast imaging with steady‐state precession (FISP) and 3 EPI, one for each possible direction of the frequency encoding (Figure 2). The simulations with the anatomic models are repeated twice, changing the thermal properties of the filling material.

For each exposure scenario, the total power deposited within the implant (*P*) and the maximum temperature increase after 900 s (Δ*T*_max,900s_) are computed. By denoting the area of the external surface of the metallic implant with the symbol *S*, the slope *m* of the linear regression between Δ*T*_max,900s_ and the ratio *P*/*S* is accounted to associate a temperature increase with the total power through a simple fit. The use of the quantity *P*/*S* is motivated by the significant influence of the implant surface on the thermal exchange (the smaller the external surface *S*, the lower the heat exchange).

Finally, to estimate the correction coefficient needed to translate in vitro results to in vivo results, the ratio between the coefficient *m* obtained simulating in vivo data (*m*_vivo_) and the one obtained in vitro (*m*_vitro_) is computed.

### 3.4 Testing of the procedure

To verify the relevance of the anisotropic index of stress *I*_aniso_(**x**) described in Equation (4) with the weighting coefficients defined in Equation (5), some in silico test cases are performed exciting a tubular GC system (Figure 1) with a 3D FISP and a EPI pulse sequence (Figure 2) where the frequency encoding is along the *x* direction (EPI‐X). The thermal effects induced by the presence of a hip, a knee, and a shoulder implant embedded in the ASTM phantom are then evaluated.

The test cases consist of 2 steps. First, the weighting coefficients are determined by a set of virtual experiments, in which the eddy currents induced in the implant by uniform magnetic fields of 1 mT at 100 Hz are computed with a validated FEM–BEM numeric method^28^, ^35^ (Supporting Information Appendix A). Next, Δ*T*_max,900s_ is estimated by simulating the implant placed within the phantom and exposed to the actual magnetic field generated during the execution of the 3D FISP and the EPI‐X pulse sequences. The simulation is repeated moving the phantom with the implant in a number of different positions within the scanner. For each position, the result is compared with the value of the index of stress calculated in the barycenter of the implant.

### 3.5 Uncertain orientation of the implant

The orientation of the implant within the body of the patient (and therefore, within the scanner) is usually not completely known. For example, the hip implant will certainly be oriented along the longitudinal axis, but small rotations because of the patient anatomy, as well as its posture, could be present along any axis.

To include this uncertainty in the anisotropic index (Equation  [4]), a Monte Carlo approach^41^ is adopted. The orientation of the implant with respect to the reference system is sampled many times according to a defined probability distribution. The index *I*_aniso_(**x**) is computed for each sampled orientation and, in a conservative viewpoint, the maximum value is kept for each position **x**.

In the computations where the uncertain orientation is taken into account, the anisotropic index is computed with 1000 Monte Carlo samples assuming that small rotations, with an amplitude uniformly distributed between −15° and +15°, could be present along any axis. The axis of rotation is identified by a unit vector whose direction is uniformly distributed on the solid angle of the sphere.^42^

## 4 RESULTS

### 4.1 Weighting coefficient

**FIGURE 5:**
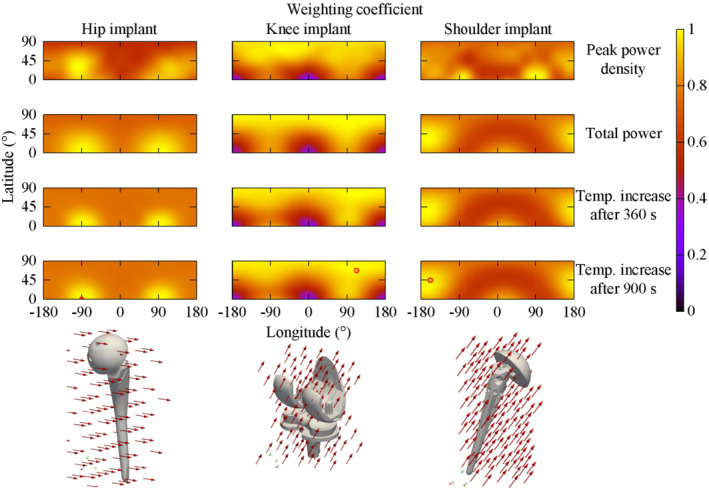
Weighting coefficient distributions in spherical coordinates for each considered implant (hip, knee, and shoulder of Yoon‐Sun model) computed with 65 virtual experiments with reference to different dosimetric quantities: peak power density (*p*_max_), total power (*P*), temperature increase after 360 s (Δ*T*_max,360s_), and after 900 s (Δ*T*_max,900s_). For the latter, the direction with the maximum coefficient is highlighted by a red circle and is represented by arrows in the 3D representation

Figure 5 shows the weighting coefficients for each considered implant and with reference to different dosimetric quantities. The 65 virtual experiments, conducted for each implant with a uniform magnetic field of 1 mT at 100 Hz varying only its orientation, lead to a variability of the dosimetric quantities in the ranges:

- from 12.4 W/m^3^ to 39.1 W/m^3^ (hip), from 5.39 W/m^3^ to 66.7 W/m^3^ (knee), and from 17.0 W/m^3^ to 48.5 W/m^3^ (shoulder) for *p*_max_;
- from 89 μW to 171 μW (hip), from 13.3 μW to 107 μW (knee), and from 42 μW to 109 μW (shoulder) for *P*;
- from 137 μK to 238 μK (hip), from 12 μK to 131 μK (knee), and from 77 μK to 221 μK (shoulder) for Δ*T*_max,360 s_; and
- from 205 μK to 358 μK (hip), from 18 μK to 187 μK (knee), and from 118 μK to 334 μK (shoulder) for Δ*T*_max,900 s_.

These values can be rescaled quadratically with respect to the magnetic field intensity and, by neglecting the skin effect, with respect to the frequency.

### 4.2 From in vitro to in silico results

**FIGURE 6:**
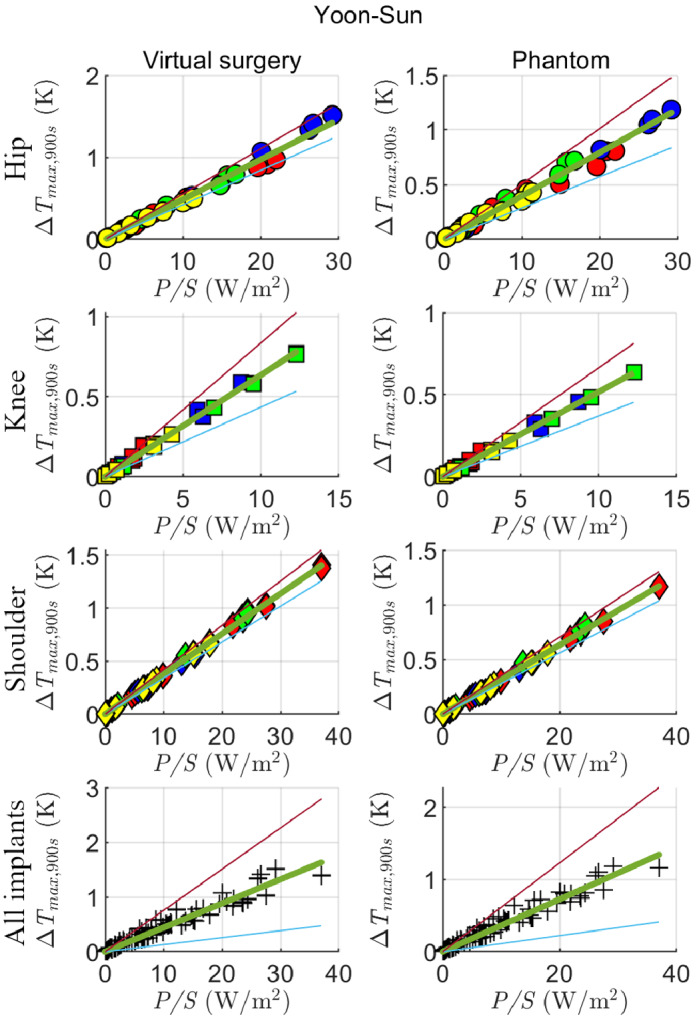
Scatter plots of Δ*T*_max,900s_ versus *P*/*S* (ratio between the total power deposited inside the implant and the external surface of the implant itself) for Yoon‐Sun model with each considered implant (first column), compared with the corresponding cases in phantom (second column). The color is representative of the considered sequence: EPI‐X (red), EPI‐Y (blue), EPI‐Z (green), 3D FISP (yellow). The results obtained combining all implants together are reported in the last row. The linear fits (green lines) are depicted together with lower (cyan) and upper (magenta) lines that include 95% of data

A linear regression between Δ*T*_max,900s_ and *P*/*S* is computed for each implanted model. The results obtained with the prostheses implanted in the Yoon‐Sun model are reported in Figure 6 (see also Supporting Information Figures S1 and S2 for the entire set of results). For each case, the plots include 72 data (9 positions, 2 filling materials, 4 pulse sequences) or 36 data (9 positions, 4 pulse sequences) for virtual surgery and phantom, respectively. The linear fitting leads to the coefficients *m* collected in Table 2, together with a range Δ*m* such that the lines with slope *m* − Δ*m* and *m* + Δ*m* include 95% of the scattered data between them.

**TABLE 2:** Coefficients of the linear regressions computed for each implanted model and ratio between the coefficients computed in vivo outcomes, and those computed in vitro

	Yoon‐Sun				Glenn				Yoon‐Sun + Glenn
	Hip	Knee	Shoulder	All	Hip	Knee	Shoulder	All	All
*m*_vivo_ (mK/[W/m^2^])	49 ± 7	64 ± 20	38 ± 4	44 ± 31	45 ± 12	60 ± 15	51 ± 11	48 ± 25	46 ± 27
With ASTM phantom properties									
*m*_vitro_ (mK/[W/m^2^])	40 ± 11	52 ± 15	32 ± 4	36 ± 25	42 ± 13	54 ± 13	47 ± 8	44 ± 21	41 ± 22
*m*_vivo_/*m*_vitro_	1.23	1.23	1.20	1.22	1.07	1.11	1.09	1.09	1.12

Similar values of *m* are obtained for analogous implants between Glenn and Yoon‐Sun, with the exception of the shoulder implant, that shows *m*_vivo_ = 51 ± 11 mK/(W/m^2^) for Glenn and *m*_vivo_ = 38 ± 4 mK/(W/m^2^) for Yoon‐Sun. The knee implants always show the largest values of *m*, as well as the largest uncertainties. The ratio between the slopes obtained in vivo (*m*_vivo_) and those obtained in vitro (*m*_vitro_) ranges between 1.20 and 1.30 for Yoon‐Sun and from 1.04 to 1.10 for Glenn, with an average of 1.16 grouping all data together. Therefore, the in vitro analysis with the ASTM gel properties always underestimates the in vivo results and needs a correction coefficient to provide conservative estimates.

### 4.3 Index of stress and test case validation

The symmetric and positive semi‐definite matrix **Q** used to compute the index of stress is evaluated for the considered 3D FISP and EPI‐X pulse sequences (Figure 2). The matrix **Q** is about

(8) $$\begin{array}{c} Q/{\left(T/m/s\right)}^{2}=\left(\begin{array}{ccc} 2888 & -16 & -448\\ -\;16 & 1166 & 0\\ -\;448 & 0 & 3150 \end{array}\right), \end{array}$$

for the 3D FISP pulse sequence, and

(9) $$\begin{array}{c} Q/{\left(T/m/s\right)}^{2}=\left(\begin{array}{ccc} 10928 & 60 & -6\\ 60 & 161 & -1\\ -\;6 & -1 & 698 \end{array}\right), \end{array}$$

for the EPI‐X pulse sequence. Other choices of the frequency encoding direction give rise to a proper permutation of the matrix rows and columns. The values in the diagonal of **Q** suggest that the 3 signals have a comparable heating potential in the 3D FISP pulse sequence, whereas only the frequency encoding direction has some relevance in the EPI pulse sequence.

**FIGURE 7:**
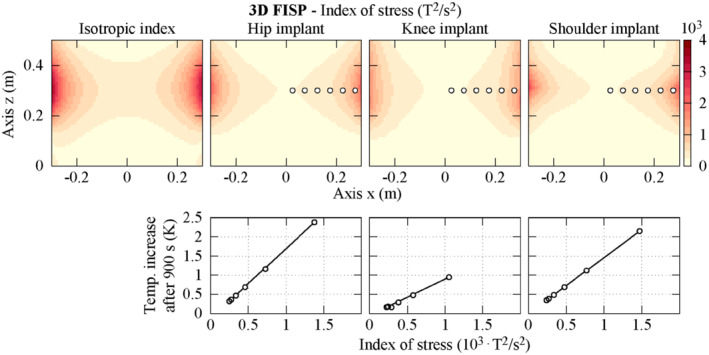
In the first row, spatial distribution in the plane *y* = 0 of the index of stress associated to the heating induced by the magnetic field generated by a tubular gradient coil system during the application of a 3D FISP pulse sequence. The isotropic index (Equation [3]) and the anisotropic index (Equation [4]) for 3 implants with weighting coefficient estimated from the peak temperature increase after 900 s are reported. In the second row, for each implant, the correlation between the anisotropic index of stress and the peak temperature increase after 900 s is shown by evaluating them when the implant is located in the position denoted by the white circles in the color maps. The considered implants are those implanted in the Yoon‐Sun model

Figure 7 shows the distribution of the isotropic index of stress *I* defined in Equation (3) related to the magnetic field generated by a tubular GC system (Figure 1) during the execution of the 3D FISP pulse sequence (the distribution for the EPI‐X sequence is reported in Support Information Figure S3). The same figure also presents the distribution of the anisotropic index of stress *I*_aniso_ defined in Equation (4) with weighting coefficients estimated from Δ*T*_max,900s_ for the 3 considered implants. The maximum value of *I* in the analyzed plane is equal to ∼3600 T^2^/s^2^ for the 3D FISP and ∼8800 T^2^/s^2^ for the EPI‐X. The index *I*_aniso_ reaches lower maximum values, equal to ∼2100 T^2^/s^2^ (FISP) and ∼5100 T^2^/s^2^ (EPI‐X) for the hip and the knee implants and ∼3000 T^2^/s^2^ (FISP) and ∼8000 T^2^/s^2^ (EPI‐X) for the shoulder implant.

For each implant, the correlation between *I*_aniso_ and Δ*T*_max,900s_ computed by simulating the implant exposed to the actual non‐uniform magnetic field generated by the GC system when located in different positions is reported in Figure 7. For this analysis, the barycenter of each implant is moved in the plane *y* = 0 along the *x*‐axis (from 0.025 m to 0.275 m in 0.05 m steps) at the quote *z* = 0.3 m. The reported plots put in evidence the linear correlation between the 2 quantities. The angular coefficients of the linear regressions with the 3D FISP pulse sequence are ∼1.8 mK/(T^2^/s^2^), ∼0.9 mK/(T^2^/s^2^), and ∼1.5 mK/(T^2^/s^2^) for the hip, knee, and shoulder implants, respectively. When the EPI‐X pulse sequence is used, the coefficients are ∼2.0 mK/(T^2^/s^2^), ∼1.1 mK/(T^2^/s^2^), and ∼1.5 mK/(T^2^/s^2^).

### 4.4 Regions of exclusion

The safety limit on the index of stress is determined for each implant with reference to Δ*T*_max,900s_ in the homogeneous ASTM phantom. This approach leads to a threshold in the index of stress equal to ∼550 T^2^/s^2^, ∼1050 T^2^/s^2^, and ∼590 T^2^/s^2^ for the hip, knee, and shoulder implant, respectively, not to overcome the safety limit of 1 K after 900 s.

**FIGURE 8:**
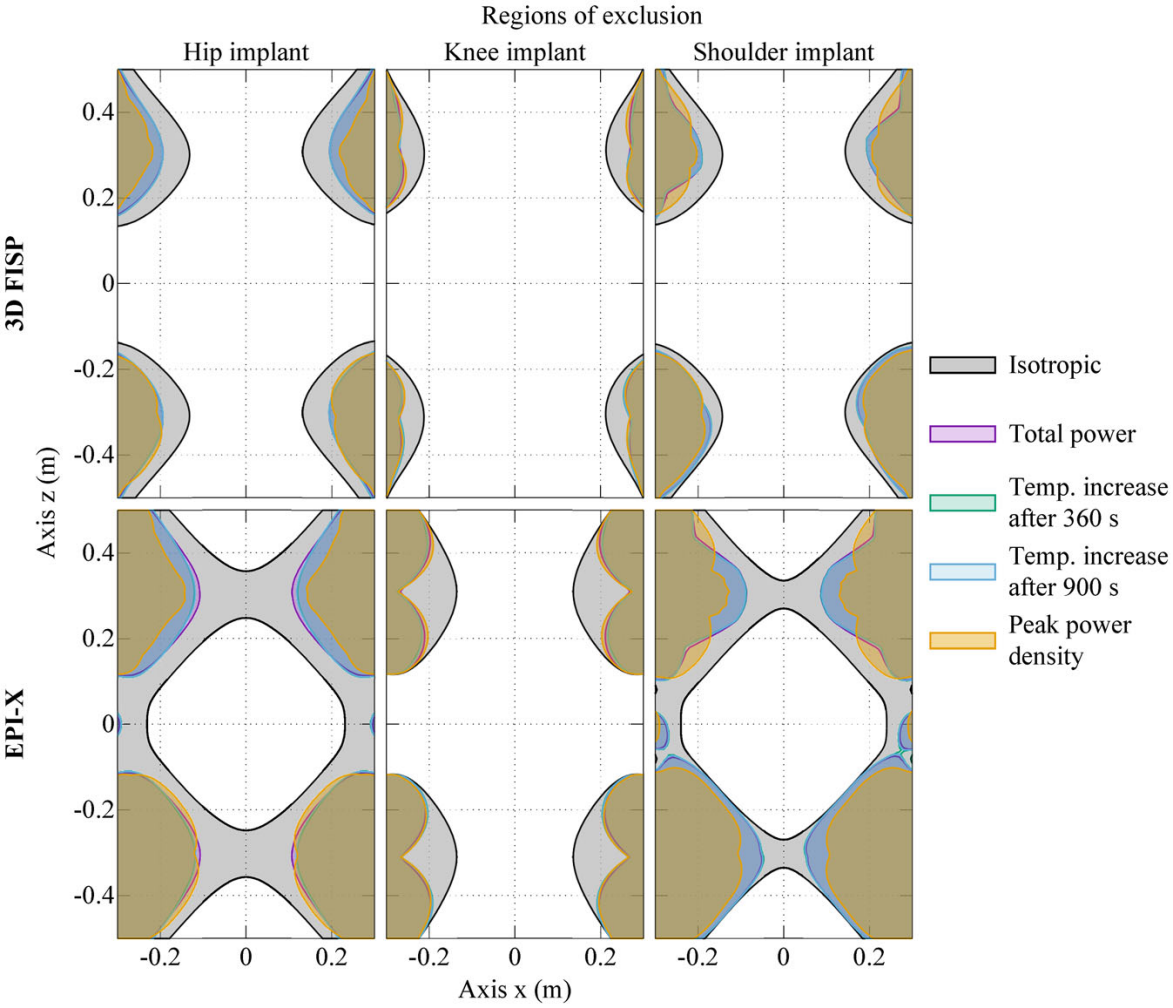
The colored areas represent the regions of exclusion for 3 implants during a 3D FISP (first row) and an EPI‐X (second row) pulse sequence executed in a tubular gradient coil system. These correspond to the positions where the implants should not be located during the exam to avoid a temperature increase, induced by the gradient fields, larger than 1 K after 900 s. For each implant, the regions are computed based on both the isotropic index of stress (Equation [3]) and the anisotropic index (Equation [4]), with weighting coefficients estimated from different dosimetric quantities. The uncertain orientation of the implant is also accounted. The considered implants are those implanted in the Yoon‐Sun model

Figure 8 shows the contour lines of the threshold value in the maps reporting the distribution of the index of stress during a 3D FISP and an EPI‐X pulse sequences (Figure 2) executed by a tubular GC system (Figure 1). The index of stress is computed with the Monte Carlo method assuming an uncertain orientation of the implant. This procedure allows to identify the regions of exclusion, where the implant should not be located during the examination to avoid a temperature increase after 900 s larger than 1 K because of the GC‐induced heating. For each implant, the regions of exclusion are computed with reference to *I* and to *I*_aniso_ with weighting coefficients estimated from the different dosimetric quantities.

The extension of the regions of exclusion can be controlled by changing the parameters of the acquired images to reduce the stress in the GC waveforms. As an example, 2 variations of the EPI‐X sequence are investigated in in the Supporting Information Appendix B.

## 5 DISCUSSION

Currently, the ISO/TS 10974 prescribes to test the implant using tabulated values of the root mean square of the time derivative of the magnetic field (*dB*/*dt*) generated by the GCs, according to values commonly adopted in clinical scanners. This reference inevitably results in a strong relation between the testing phase of the device and the state‐of‐art of the GC technology, which is under rapid evolution. The presented procedure aims to overcome this limitation by decoupling the implant testing process, to be performed in the laboratory under defined exposure conditions, from the translation of the results into the clinical practice, whatever GC system and pulse sequence are used. The schematic summary presented in Figure 4 helps highlighting it.

In this way, the role of the implant manufacturer and testing laboratory is clearly separated from the role of the scanner producer. The former can experimentally characterize the implant (Figure 3), whereas the latter, based on implant characterization and index of stress, can determine the regions of exclusion (Figure 8) to be adopted by the radiologist during the MRI session.

The proposed procedure, being based on an anisotropic index of stress that takes into account the orientations of the implant within the scanner, leads to restrictions that are not unnecessarily strict. This is evident comparing the regions of exclusion evaluated with the isotropic and the anisotropic indexes (Figure 8). The isotropic index leads to regions of exclusion that contain the regions obtained with any anisotropic index. The anisotropic region grows when the uncertainty in the implant orientation increases, up to the point where it coincides with the isotropic region in the case of complete lack of knowledge. It is worth noting that the regions of exclusion, computed assuming the head of the patient toward the positive *z*‐axis, are not symmetric with respect to the plane *z* = 0.

It must be remarked that the evaluation of the regions of exclusion is conducted assuming a uniform distribution of the magnetic field within the implant. Therefore, it is enough that a portion of the implant falls in a region of exclusion to make the examination risky. In this case, a complete analysis of the implant heating could be conducted as a sort of Tier 2 approach.

The anisotropic index and the related regions of exclusion depend on the selected dosimetric quantity. However, whereas *P*, Δ*T*_max,360s_ and Δ*T*_max,900s_ show quite similar results, those obtained with *p*_max_ are significantly different. In particular, the results obtained with Δ*T*_max,360s_ are not distinguishable from those obtained with Δ*T*_max,900s_. Because Δ*T*_max,900s_ quantifies the actual physical effect at the basis of the selected safety limits, *p*_max_ cannot be considered a good dosimetric quantity for the GC‐induced heating. On the other hand, the total power *P* correlates well with Δ*T*_max,900s_, suggesting its suitability as dosimetric quantity.

A testing procedure measuring *P* through electrical measurements could have practical advantages with respect to the measurement of the maximum temperature increase. If it is adopted, a reliable correlation coefficient between *P* and Δ*T*_max,900s_ should be determined for any implant. According to Table 2, it can be identified with a relative variability ranging from 10% to 30% taking into account differences due to implant positioning and adopted sequence.

Finally, to identify the threshold on the dosimetric quantity, the results obtained in vitro and in vivo must be correlated. Table 2 shows that, referring to the gel recommended by the ASTM standard, the ratio between in vivo and in vitro results varies from 1.09 to 1.23, being always higher for the former despite the absence of thermoregulation effects. This outcome is not surprising, because the thermal properties of the gel are quite similar to those of the muscle,^40^ whereas the implants, where the energy is directly deposited by the gradient fields, are mainly surrounded by bone. Because the thermal conductivity of the bone is significantly lower than that of the gel,^40^ the bone surrounding the implant acts like a thermal insulator, producing a larger temperature increase. The weak blood perfusion of the bone tissues is not enough to hinder this effect at the interface between bone and metal. New tissue mimicking materials could be designed to reproduce better the in vivo results with phantom measurements.

## 6 CONCLUSIONS

A contribution to future extension of the testing procedure of implants exposed to the gradient field of MRI scanner is described. The main attempt of the proposed approach is to decouple the testing phase of the device and the translation of the result in the clinical practice, with the aim of extending the applicability of the device characterization to a variety of clinical operating conditions (adopted hardware, clinical sequences, etc.). By integrating the results of the laboratory testing with technical information of the scanner producer, practical guides could be provided to the radiologist on a case‐by‐case basis. Moreover, the procedure for the definition of the index of stress has been developed considering the actual shape of orthopedic implants to avoid unnecessarily strict limits introduced by the use of simplified shapes.

The procedure has been presented adopting a theoretical approach based on numeric simulations, to put in evidence merits and drawbacks. A future step will be to implement this methodology into an experimental procedure and to compare its indications with measurements carried out on realistic setups. Finally, the procedure needs to be validated against entire MRI protocols, consisting of several pulse sequences.

## Supporting information


**Figure S1.** Scatter plots of Δ*T*_max,900s_ versus *P*/*S* (ratio between the total power deposited inside the implant and the external surface of the implant itself) for Glenn and Yoon‐Sun models with each considered implant (first and third columns), compared with the corresponding cases in phantom (second and fourth columns). The color is representative of the considered sequence: EPI‐X (red), EPI‐Y (blue), EPI‐Z (green), 3D FISP (yellow). The results obtained combining all implants together are reported in the last row. The linear fits are depicted together with lower and upper lines including 95% of data.
**Figure S2.** Scatter plots of Δ*T*_max,900s_ versus *P*/*S* (ratio between the total power deposited inside the implant and the external surface of the implant itself) combining all the data together. The linear fits are depicted together with lower and upper lines including 95% of data, and their slopes are reported.
**Figure S3.** In the first row, spatial distribution in the plane *y* = 0 of the index of stress associated to the heating induced by the magnetic field generated by a tubular gradient coil system during the application of an EPI‐X pulse sequence. The isotropic index Equation (3) and the anisotropic index Equation (4) for 3 implants with weighting coefficient estimated from the peak temperature increase after 900 s are reported. In the second row, for each implant, the correlation between the anisotropic index of stress and the peak temperature increase after 900 s is shown by evaluating them when the implant is located in the position denoted by the white circles in the color maps. The considered implants are those implanted in the Yoon‐Sun model.
**Figure S4.** Gradient coil setup adopted for the experiments.
**Figure S5.** Shoulder (**A**) and knee (**B**) implant with the optical fiber temperature probes positioned on their surface. The labels of the channels are reported in the pictures.
**Figure S6.** Orientation of the shoulder (**A**) and knee (**B**) implant with respect to the Cartesian reference system. Both the implants are represented here in the position denoted by the angle α=0°.
**Figure S7.** Gradient waveforms of the trapezoidal sequences used during the experiments (**A,B**) and their time derivatives (**C,D**).
**Figure S8.** Temperature increase in the shoulder implant oriented with α=0° (**A**) and α=45° (**B**) with the Z‐coil waveform.
**Figure S9.** Temperature increase in the shoulder (**A**) and the knee (**B**) implant with the X‐coil waveform.
**Figure S10.** The colored areas represent the regions of exclusion for Yoon‐Sun hip implant during the 3 pulse sequences executed in a tubular gradient coil system. These correspond to the positions where the implants should not be located during the exam to avoid a temperature increase, induced by the gradient fields, larger than 1 K after 900 s. For each sequence, the regions are computed based on both the isotropic and the anisotropic indexes of stress, with weighting coefficients estimated from different dosimetric quantities. The uncertain orientation of the implant is also accounted.
**Table S1.** GC system and amplifier setup maximum performances.
**Table S2.** Main parameters of the sequences designed to observe the influence of image parameters on the GC‐induced heating.
Appendix A.
Appendix B.
