# Supplementary material for: A contribution to MRI safety testing related to gradient‐induced heating of medical devices
Source: Magn Reson Med. 2022 Mar 28;88(2):930–44. doi: 10.1002/mrm.29235 (PMC9314691; doi:10.1002/mrm.29235)
Supplement: Supplementary file 1 — Figure S1. Scatter plots of ΔT max,900s versus P/S (ratio between the total power deposited inside the implant and the external surface of the implant itself) for Glenn and Yoon‐Sun models with each considered implant (first and third columns), compared with the corresponding cases in phantom (second and fourth columns). The color is representative of the considered sequence: EPI‐X (red), EPI‐Y (blue), EPI‐Z (green), 3D FISP (yellow). The results obtained combining all implants together are reported in the last row. The linear fits are depicted together with lower and upper lines including 95% of data. Figure S2. Scatter plots of ΔT max,900s versus P/S (ratio between the total power deposited inside the implant and the external surface of the implant itself) combining all the data together. The linear fits are depicted together with lower and upper lines including 95% of data, and their slopes are reported. Figure S3. In the first row, spatial distribution in the plane y = 0 of the index of stress associated to the heating induced by the magnetic field generated by a tubular gradient coil system during the application of an EPI‐X pulse sequence. The isotropic index Equation (3) and the anisotropic index Equation (4) for 3 implants with weighting coefficient estimated from the peak temperature increase after 900 s are reported. In the second row, for each implant, the correlation between the anisotropic index of stress and the peak temperature increase after 900 s is shown by evaluating them when the implant is located in the position denoted by the white circles in the color maps. The considered implants are those implanted in the Yoon‐Sun model. Figure S4. Gradient coil setup adopted for the experiments. Figure S5. Shoulder (A) and knee (B) implant with the optical fiber temperature probes positioned on their surface. The labels of the channels are reported in the pictures. Figure S6. Orientation of the shoulder (A) and knee (B) implant with respect to the Cartesia [file MRM-88-930-s001.pdf]

A contribution to MRI safety testing related to gradient-induced heating of medical devices

Alessandro Arduino*, Oriano Bottauscio, Mario Chiampi, Umberto Zanovello, Luca Zilberti

Istituto Nazionale di Ricerca Metrologica (INRIM), Torino, ITALY

*Corresponding author (a.arduino@inrim.it)

Supporting information

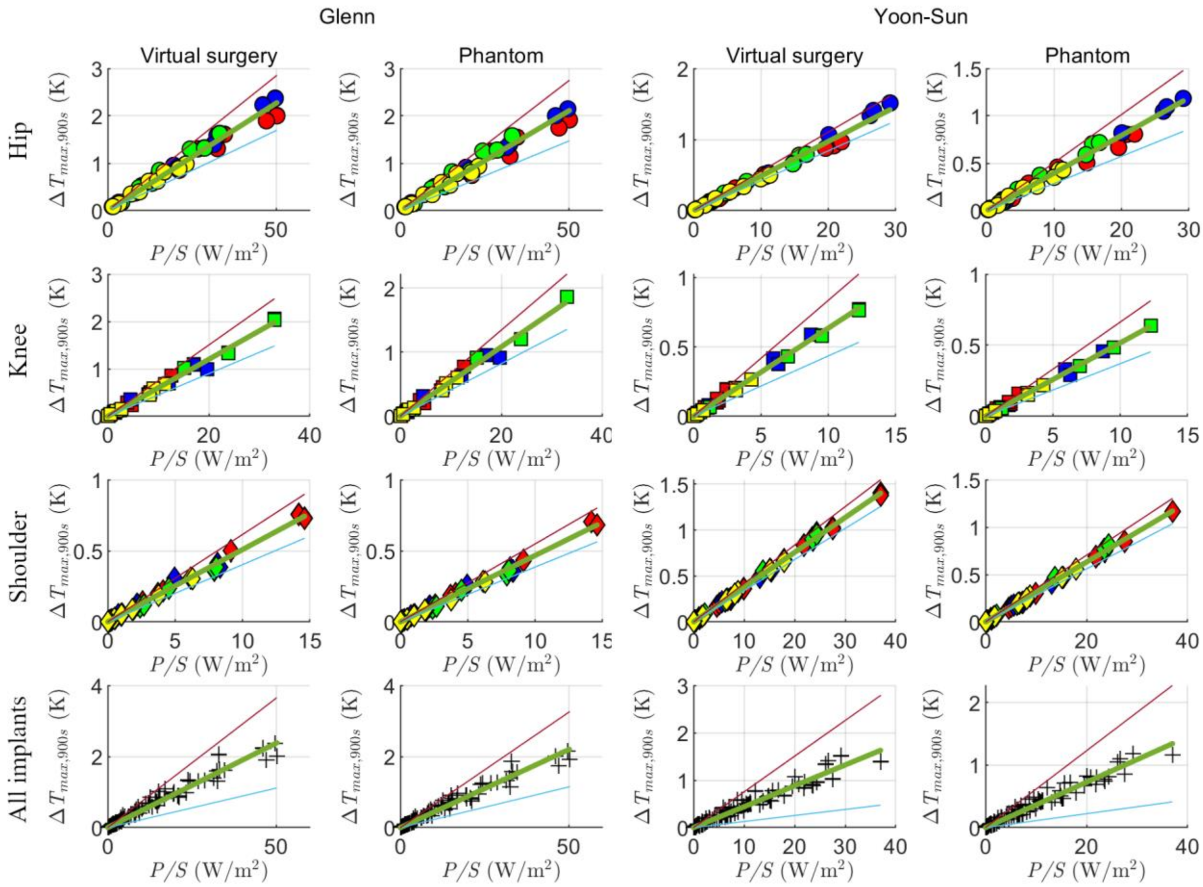

Supporting Information Figure S1. Scatter plots of $\Delta T_{\text{max,900s}}$ versus P/S (ratio between the total power deposited inside the implant and the external surface of the implant itself) for Glenn and Yoon-Sun models with each considered implant (first and third columns), compared with the corresponding cases in phantom (second and fourth columns). The color is representative of the considered sequence: EPI-X (red), EPI-Y (blue), EPI-Z (green), 3D FISP (yellow). The results obtained combining all implants together are reported in the last row. The linear fits are depicted together with lower and upper lines including 95 % of data.

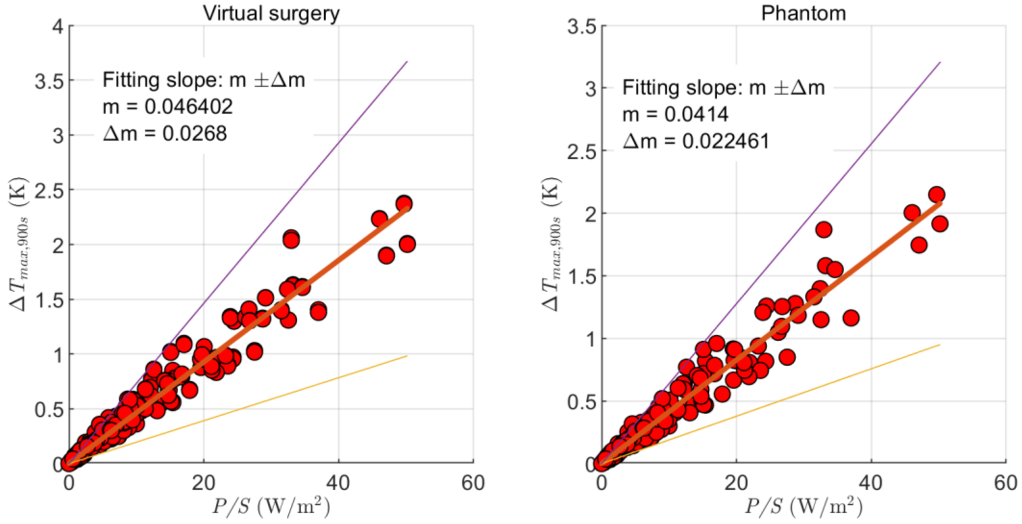

Supporting Information Figure S2. Scatter plots of $\Delta T_{\text{max},900\text{s}}$ versus P/S (ratio between the total power deposited inside the implant and the external surface of the implant itself) combining all the data together. The linear fits are depicted together with lower and upper lines including 95 % of data, and their slopes are reported.

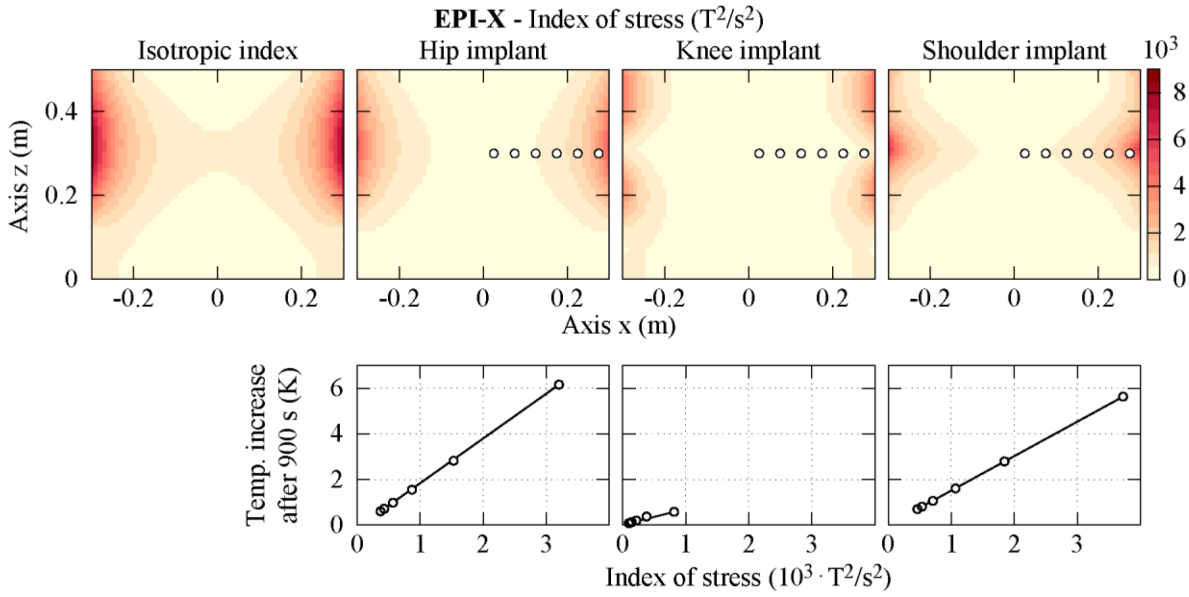

Supporting Information Figure S3. In the first row, spatial distribution in the plane $y = 0$ of the index of stress associated to the heating induced by the magnetic field generated by a tubular gradient coil system during the application of an EPI-X pulse sequence. The isotropic index (3) and the anisotropic index (4) for three implants with weighting coefficient estimated from the peak temperature increase after 900 s are reported. In the second row, for each implant, the correlation between the anisotropic index of stress and the peak temperature increase after 900 s is shown by evaluating them when the implant is located in the position denoted by the white circles in the color maps. The considered implants are those implanted in the Yoon-Sun model.

Supporting Information Appendix A

Laboratory experiments are performed with two aims: a) to validate the modelling results under different exposure scenarios; b) to give evidence of the effect of implant orientation with respect to the applied gradient field. The results are collected for two implant types considered in the article: the shoulder implant and the tibial part of the knee implant. These two implants have been chosen because of their different shapes. The hip implant is not considered, since it is similar to the shoulder one. Furthermore, a validation of the model with hip implants was already provided in previous publications[1,2].

The experiments are done using a realistic tubular gradient coil set-up (mod. Solaris-R by Nanjing Cichen Medical Technology Co., Ltd, Nanjing, China), with internal diameter of 670 mm and total length of 1501 mm. Supporting Information Figure S4 shows the laboratory set-up. The sensitivities of the three gradient coils are: 0.056 mT/m/A for the X-coil, 0.055 mT/m/A for the Y-coil, and 0.059 mT/m/A for the Z-coil. The coils are individually supplied by a IECO amplifier (MAGNET POWER SUPPLYSYSTEM - MPS-1200-350) configured to provide a maximum voltage of 400 V and a maximum current of 300 A. The maximum performances allowed by the amplifier characteristics are detailed in the Supporting Information Table S1. The amplifier is controlled by a NI PXIe 5423 waveform generator module connected to a NI PXIe-1065 chassis allowing to generate voltage signals with a sample rate ranging from 3.125 to 200 megasamples per second and a waveform size ranging from 2 samples to 64 megasamples, allowing for a reliable reproduction of common MRI GC signals. The output voltage and current monitors of the amplifiers are connected to a NI PXIe 4464 analog signal acquisition module to monitor the generated current and voltage in real time (sample rate from 100 samples per second to 204.8 kilosamples per second).

During the experiments, six calibrated optical fiber temperature probes are connected to an 8-channel AccuSens OPSense remote unit to acquire the temperature increase in different target points on the implant surface. This measurement setup has a resolution of 0.01 K. Supporting Information Figure S5 shows the two implants and the optical fiber temperature probes positioned on their surfaces. In the reference position, the two implants are oriented with respect to the Cartesian axes as shown in the Supporting Information Figure S6. The rotation angle α used in the following results indicates a counterclockwise rotation of the implant in the *xz*-plane around the *y*-axis ($\alpha = 0°$ denotes the reference position shown in the figure). The implants have been placed in almost adiabatic conditions within the scanner bore.

The two trapezoidal sequences reported in Supporting Information Figure S7, named Trap-Z and Trap-X, are used to supply the GC system. In Trap-Z (Trap-X), only the coil Z (coil X) is supplied according to a trapezoidal waveform. The sequences are repeated periodically for a total duration of 900 s.

Supporting Information Figure S8a shows the time evolution of the temperature increase on the shoulder implant with $\alpha = 0°$ when exposed to the field generated by Trap-Z in the coordinates x = 191 mm, y = 44.5 mm, z = 346 mm with respect to the isocenter, where $\mathbf{B}_3$ / $(\mathrm{T}/(\mathrm{T}/\mathrm{m})) = (0.025, 0.004, 0.327)$. The reported data are registered by the probes placed on the shoulder head (CH2, cf. Supporting Information Figure S5) and at the tip of the stem (CH1, cf. Supporting Information Figure S5). The temperature increase computed through the simulation of the experimental setup in the same positions of the two probes shows an excellent agreement between measurements and simulations. Supporting Information Figure S8b shows similar data for the shoulder implant with $\alpha = 90°$ exposed to Trap-Z in the same coordinates as before. A substantial reduction of the heating is observed with respect to the previous orientation.

---

[1] Arduino A, Bottauscio O, Brühl R, Chiampi M, Zilberti L. In silico evaluation of the thermal stress induced by MRI switched gradient fields in patients with metallic hip implant. Phys Med Biol 2019;64:245006. doi: 10.1088/1361-6560/ab5428.

[2] Bruehl R, Schwenteck T, Ittermann B, Baruffaldi F, Arduino A, Zanovello U, Zilberti L, Chiampi M, Bottauscio O. Experimental validation of simulated implant heating induced by switched gradient fields. Proc Intl Soc Mag Reson Med 29 (2021); 2288.

Supporting Information Figure S9a reports the heating of the shoulder implant with $\alpha = 45°$ and $\alpha = 90°$ exposed to the field generated by Trap-X in the coordinates *x* = 194 mm, *y* = 44.5 mm, *z* = 385 mm with respect to the isocenter, where $\mathbf{B}_1$ / $(\mathrm{T}/(\mathrm{T}/\mathrm{m})) = (0.400, 0.003, 0.012)$. For both the orientations, the temperature increase measured on the shoulder head (probe CH2, cf. Supporting Information Figure S5) is reported. Also in this case, the induced heating depends on the orientation of the implant. Finally, Supporting Information Figure S9b shows the time evolution of the temperature increase of the tibial part of the knee implant with $\alpha = 45°$ and $\alpha = 90°$ exposed to the field generated by Trap-X in the same coordinates as before. For both the orientations, the data reported are registered by the probe on the boundary of the tibial plate (CH0, cf. Supporting Information Figure S5), where the heating is maximized. For both the implants, the agreement between measurements and simulations is good and the dependence of the heating on the orientation of the implant is demonstrated.

The two probes on the shoulder implant show that different regions of the implant heat up in different ways. This happens because of the complex implant shape and the consequent distribution of the eddy currents within the object. Thus, besides the diffusion of the heating from the implant towards the surrounding environment, a sensible heat diffusion within the implant itself takes place during the exposure to the switching gradient fields.

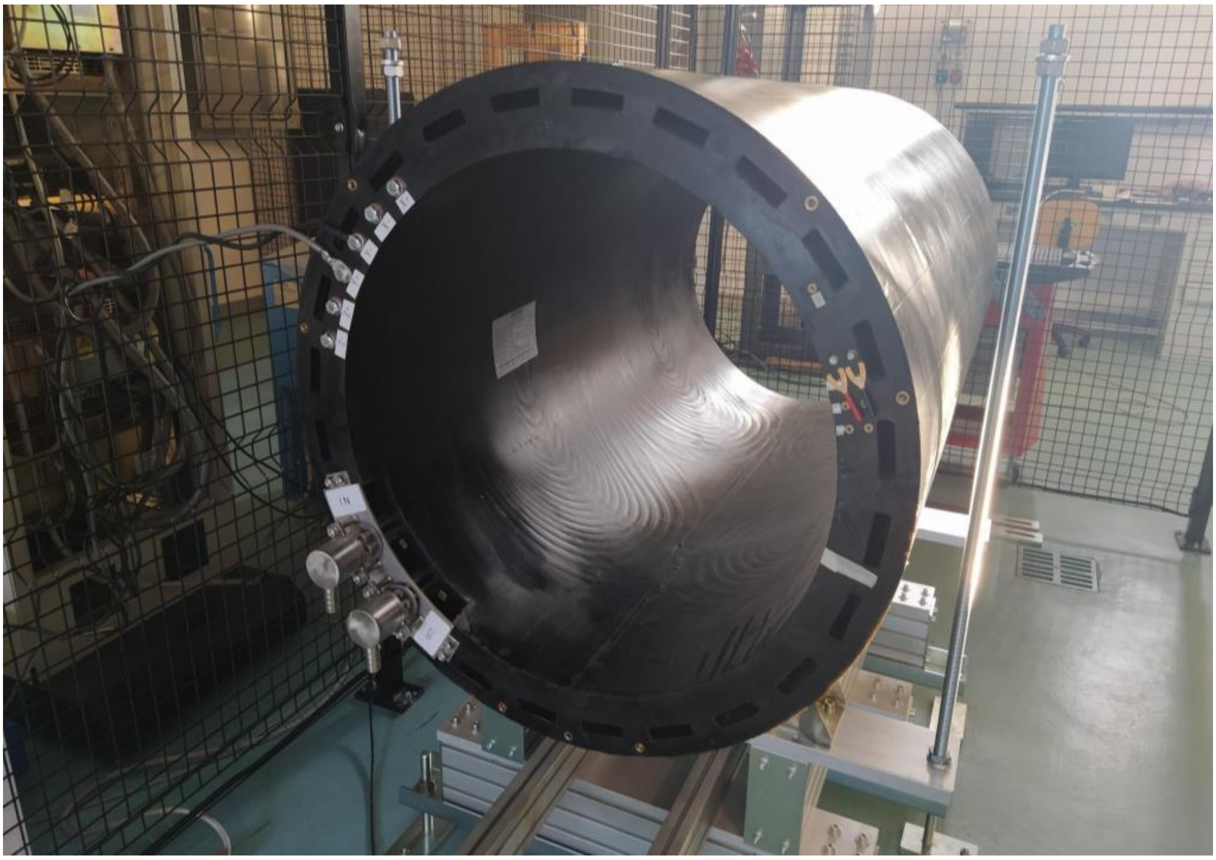

Supporting Information Figure S4. Gradient coil setup adopted for the experiments

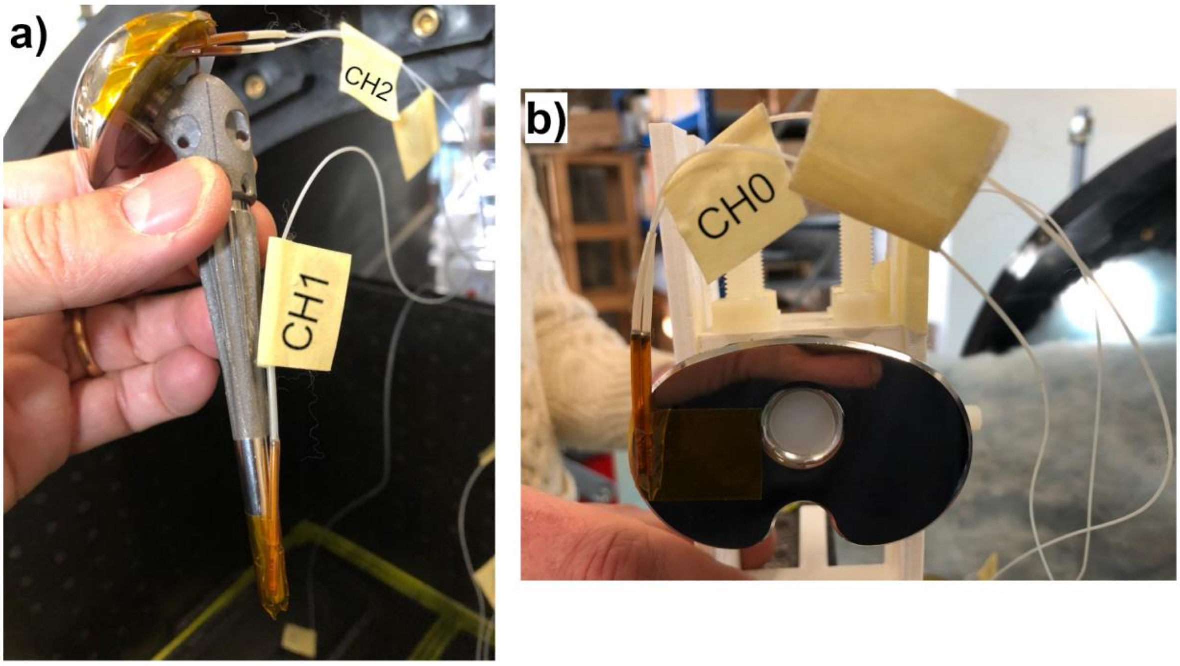

Supporting Information Figure S5. Shoulder (**a**) and knee (**b**) implant with the optical fibre temperature probes positioned on their surface. The labels of the channels are reported in the pictures.

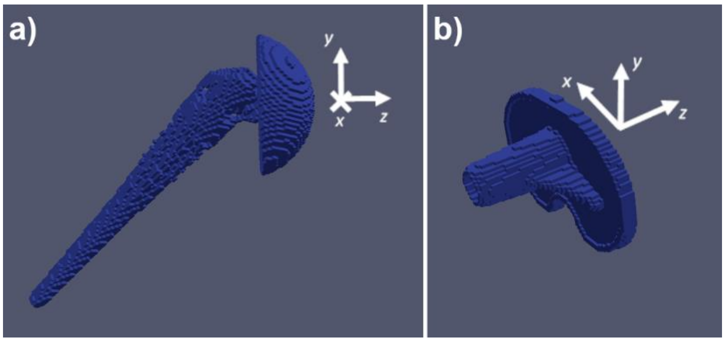

Supporting Information Figure S6. Orientation of the shoulder (**a**) and knee (**b**) implant with respect to the Cartesian reference system. Both the implants are represented here in the position denoted by the angle $\alpha = 0°$.

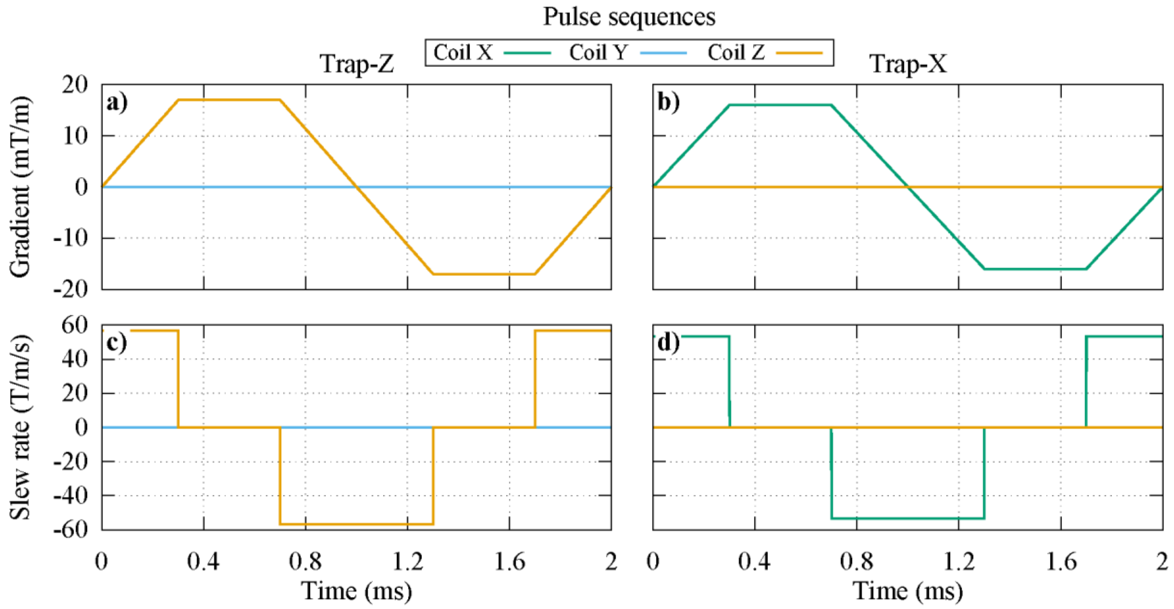

Supporting Information Figure S7. Gradient waveforms of the trapezoidal sequences used during the experiments (**a, b**) and their time derivatives (**c, d**).

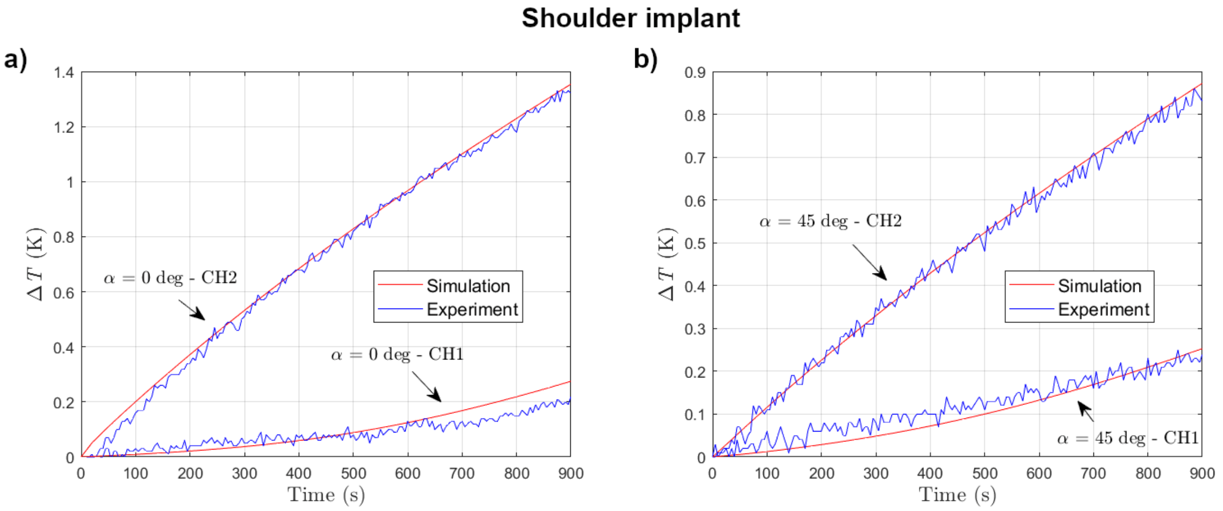

Supporting Information Figure S8. Temperature increase in the shoulder implant oriented with $\alpha = 0°$ (**a**) and $\alpha = 45°$ (**b**) with the Z-coil waveform.

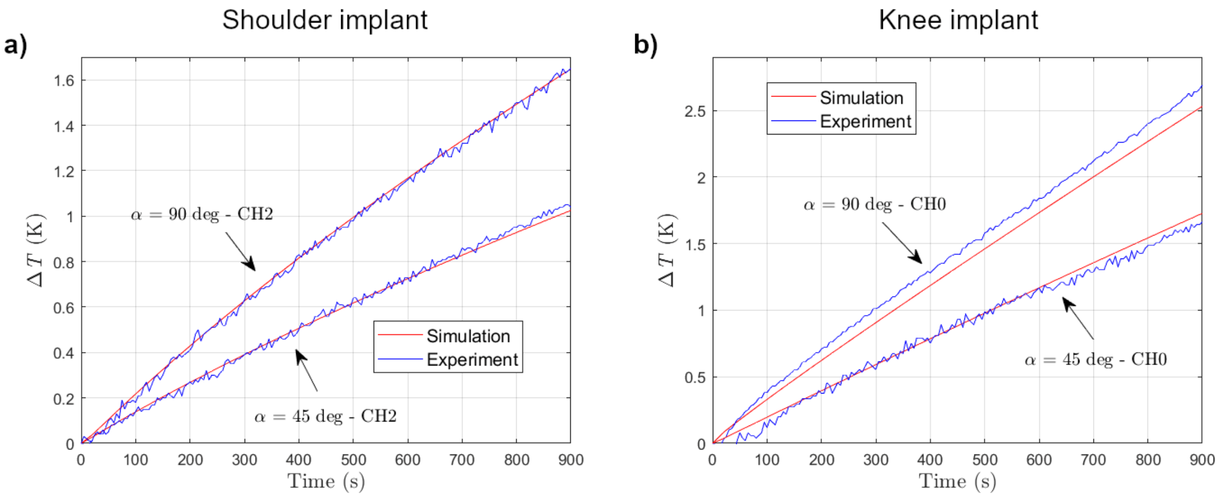

Supporting Information Figure S9. Temperature increase in the shoulder (**a**) and the knee (**b**) implant with the X-coil waveform.

	X Coil	Y Coil	Z Coil
**Maximum gradient (mT/m)**	16.75	16.5	17.7
**Maximum slew rate (T/m/s)**	62.55	61.28	62.93

Supporting Information Table S1. GC system and amplifier setup maximum performances.

Supporting Information Appendix B

In order to observe the effect of the image parameters, like the image size or the field of view, on the index of stress, two variations of the EPI-X sequence are designed and compared. The sequences are designed using the open-source software Pulseseq (https://pulseq.github.io/) and their main parameters are listed in Supporting Information Table S2.

The first variation of the EPI-X sequence (EPI-X-v1) is obtained starting from the original sequence by doubling the image size. The other variation (EPI-X-v2) is obtained by reducing the field of view together with the readout bandwidth. To be consistent with the fixed maximum gradient amplitude and slew rate, a change of the repetition time was necessary.

The different gradient waveforms of the pulse sequences are responsible for different distributions of the index of stress. With respect to EPI-X, in EPI-X-v1 the length of the flat top in the gradient signal that performs the readout is doubled, whereas the number and duration of the switches within each period of the signal is kept the same. Therefore, the same energy is deposited in the implant by the eddy currents induced by the switches, but in a longer time. This is equivalent to the introduction of idle intervals in the sequence (i.e., a reduction of its duty-cycle) and leads to a lower index of stress. Differently, the lower readout bandwidth of EPI-X-v2 makes it unnecessary to reach the maximum gradient during the readout. The shorter rise/fall times, combined to the doubled flat top length with respect to EPI-X, lead to a decrease of the index of stress, also in this case. As a consequence, both the sequence variations are expected to heat less than the original EPI-X sequence. As a drawback, a lower SNR is expected from the EPI-X-v1, because of smaller pixels and longer acquisition time, and wrap-around artefacts can arise in EPI-X-v2 due to undersampling.

The matrices **Q** associated to each sequence are reported below and corroborate these observations. The off-diagonal components of the matrices are almost negligible with respect to the largest diagonal component, which can be used as the term of comparison between the different sequences. The largest value is found for the original EPI-X, whereas smaller values are associated to the sequence variations:

EPI-X: $$\mathbf{Q}\ /\ (\mathrm{T/m/s})^2 = \begin{pmatrix} 10928 & 60 & -6 \\ 60 & 161 & -1 \\ -6 & -1 & 698 \end{pmatrix},$$

EPI-X-v1: $$\mathbf{Q}\ /\ (\mathrm{T/m/s})^2 = \begin{pmatrix} 8295 & 59 & 41 \\ 59 & 77 & 42 \\ 41 & 42 & 691 \end{pmatrix},$$

EPI-X-v2: $$\mathbf{Q}\ /\ (\mathrm{T/m/s})^2 = \begin{pmatrix} 6364 & 84 & 61 \\ 84 & 179 & 63 \\ 61 & 63 & 1284 \end{pmatrix}.$$

Supporting Information Figure S10 shows the regions of exclusion for the Yoon-Sun hip implant estimated with the isotropic and the anisotropic stress indexes, as described in the main text. The different extension of such regions indicates a lower risks associated with the sequence variations.

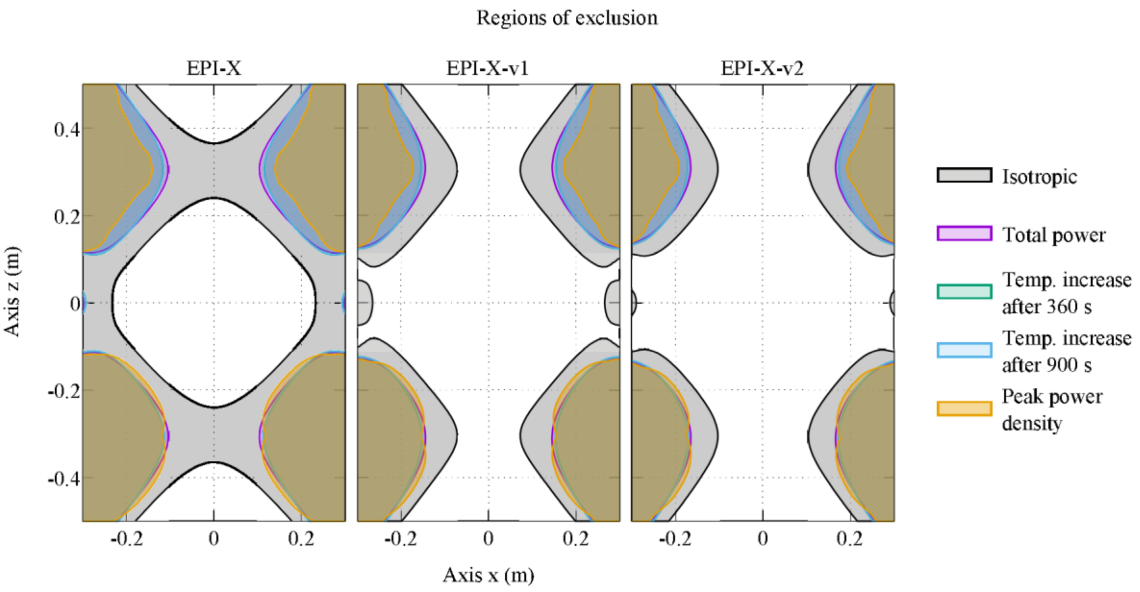

Supporting Information Figure S10. The colored areas represent the regions of exclusion for Yoon-Sun hip implant during the three pulse sequences executed in a tubular gradient coil system. These correspond to the positions where the implants should not be located during the exam to avoid a temperature increase, induced by the gradient fields, larger than 1 K after 900 s. For each sequence, the regions are computed on the basis of both the isotropic and the anisotropic indexes of stress, with weighting coefficients estimated from different dosimetric quantities. The uncertain orientation of the implant is also accounted.

	**EPI-X**	**EPI-X-v1**	**EPI-X-v2**
**Echo time (ms)**	22	55	27
**Repetition time (ms)**	43	107	53
**Image size**	64 × 64	128 × 128	64 × 64
**Field of view (mm)**	310 × 310	310 × 310	210 × 210
**Slew rate (T/m/s)**	160	160	160
**Maximum gradient (mT/m)**	23	23	23
**Bandwidth readout (kHz)**	300	300	150

Supporting Information Table S2. Main parameters of the sequences designed to observe the influence of image parameters on the GC-induced heating.
